# Arterially Perfused Neurosphere-Derived Cells Distribute Outside the Ischemic Core in a Model of Transient Focal Ischemia and Reperfusion *In Vitro*

**DOI:** 10.1371/journal.pone.0002754

**Published:** 2008-07-23

**Authors:** Chiara Pastori, Laura Librizzi, Gian Luca Breschi, Cristina Regondi, Carolina Frassoni, Ferruccio Panzica, Simona Frigerio, Maurizio Gelati, Eugenio Parati, Maria Grazia De Simoni, Marco de Curtis

**Affiliations:** 1 Unità di Neurofisiologia ed Epilettologia Sperimentale, Fondazione Istituto Neurologico Carlo Besta, Milan, Italy; 2 Unità di Neurofisiopatologia ed Epilettologia diagnostica, Fondazione Istituto Neurologico Carlo Besta, Milan, Italy; 3 SDS Neurobiologia e Terapie Neuroriparative, Fondazione Istituto Neurologico Carlo Besta, Milan, Italy; 4 Istituto di Ricerche Farmacologiche Mario Negri, Milano, Italy; 5 Neurology Residency School, University of Milano-Bicocca, Monza, Italy; Columbia University, United States of America

## Abstract

**Background:**

Treatment with neural stem cells represents a potential strategy to improve functional recovery of post-ischemic cerebral injury. The potential benefit of such treatment in acute phases of human ischemic stroke depends on the therapeutic viability of a systemic vascular delivery route. In spite of the large number of reports on the beneficial effects of intracerebral stem cells injection in experimental stroke, very few studies demonstrated the effectiveness of the systemic intravenous delivery approach.

**Metodology/Principal Findings:**

We utilized a novel *in vitro* model of transient focal ischemia to analyze the brain distribution of neurosphere-derived cells (NCs) in the early 3 hours that follow transient occlusion of the medial cerebral artery (MCA). NCs obtained from newborn C57/BL6 mice are immature cells with self-renewal properties that could differentiate into neurons, astrocytes and oligodendrocytes. MCA occlusion for 30 minutes in the *in vitro* isolated guinea pig brain preparation was followed by arterial perfusion with 1×10^6^ NCs charged with a green fluorescent dye, either immediately or 60 minutes after reperfusion onset. Changes in extracellular pH and K^+^ concentration during and after MCAO were measured through ion-sensitive electrodes.

**Conclusion/Significance:**

It is demonstrated that NCs injected through the vascular system do not accumulate in the ischemic core and preferentially distribute in non-ischemic areas, identified by combined electrophysiological and morphological techniques. Direct measurements of extracellular brain ions during and after MCA occlusion suggest that anoxia-induced tissue changes, such as extracellular acidosis, may prevent NCs from entering the ischemic area in our *in vitro* model of transitory focal ischemia and reperfusion suggesting a role played by the surrounding microenviroment in driving NCs outside the ischemic core. These findings strongly suggest that the potential beneficial effect of NCs in experimental focal brain ischemia is not strictly dependent on their homing into the ischemic region, but rather through a bystander mechanism possibly mediated by the release of neuroprotective factors in the peri-infarct region.

## Introduction

Neuronal stem cells are immature cells with self-renewal potentials that are able to differentiate into neurons, astrocytes and oligodendrocytes [1]–[2]. Brain transplantation and the induction of the proliferation of neural stem and precursor cells represent a potential strategy to improve the functional deficits caused by CNS injury [3]–[4]. Experimental findings support the hypothesis that treatment with various types of stem cells and other immature cells identifiable as staminal in nature may be beneficial in repairing the brain damage induced by an ischemic insult [5]–[6]. Stem cells grafted by either intracerebral or intracerebroventricular local application show the tendency to migrate from their site of injection toward the peri-ischemic brain region, where they are believed to replace the cells that have died [7]–[8]–[9]. Similar results were showed in the few studies in which neural stem cells were transplanted by intravenous injection [10]–[11]–[12]. The survival of stem cells and the mechanisms involved in their neuroprotective effects was not consistently evaluated in these experimental studies (see [6]. A recent report demonstrated that the functional improvement of open field performances and the neuronal survival in a mouse model of transient occlusion of the medial cerebral artery (MCA) is independent on the persistence of intracerebroventricularly injected neurosphere-derived cells (NCs) in the brain parenchyma [13]. These findings strongly suggest that the favorable effects of NCs may be due to the release of neuroprotective factors in the ischemic microenvironment, rather then to the replacement of damaged neurons.

The likelihood to use stem cells as potential therapy in human stroke depends on the availability of a minimally invasive direct route of delivery. It is obvious that intracerebral application of stem cells is impracticable in a clinical setting, at least with the presently available technology, whereas systemic injection via a venous access is the most favorable. Even though this issue is crucial for translating experimental findings into clinical practice, very few experimental studies utilize this delivery route to evaluate the efficacy of stem cell treatment on ischemic brain damage [10]–[11]. In addition, the possible therapeutic use of stem cells in cerebral ischemia depends on the identification of the ideal time window suitable for an effective treatment. This information is still missing.

The main question addressed in the present study is to investigate whether NCs show a chemo-attraction for the ischemic region in the first few hours that follow a transient cerebral ischemia. To answer to this question, we recently developed a model of transient MCA occlusion in the in whole guinea pig brain maintained in vitro by arterial perfusion through the resident vascular system, isolated en block with the brain [14]. Previous reports have demonstrated that the complex interactions between extracellular and intracellular compartments [15]–[16], the vascular compartment [17]–[18] and the blood–brain barrier [19]–[20]–[21] are functionally and structurally preserved for several hours in the isolated guinea pig brain. In this preparation the access to the major arterial branches that originate from the Willis circle is facilitated because of the structural preservation and exposure of the resident arterial system and therefore MCA occlusion can be easily performed. The transient MCA occlusion and subsequent reperfusion in this experimental preparation represents a close to *in vivo* condition, in which the electrophysiological correlates of the early events associated with the ischemic process can be dynamically analyzed.

We demonstrate that NCs do not target the ischemic region during the 2 hours reperfusion that follows a 30-minute MCA occlusion. Our findings suggest that the acidic microenvironment in the ischemic core may be responsible for the tissue rejection of NCs arterially injected just after MCA reperfusion.

## Results

**Figure 1:**
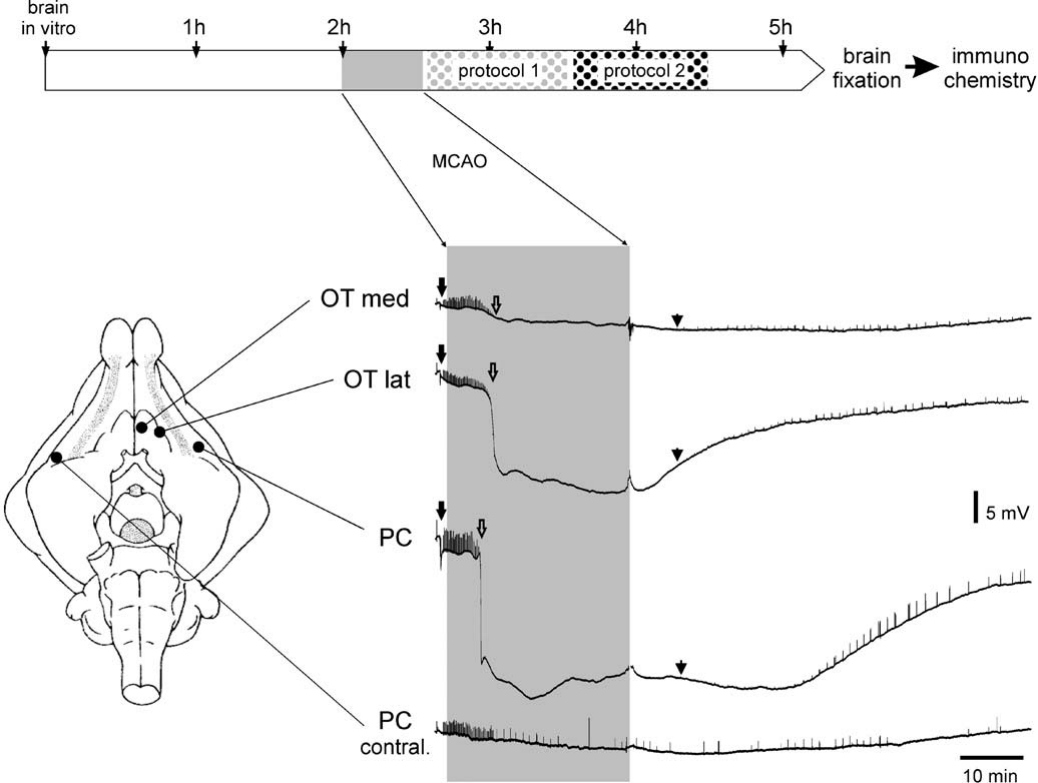
Experimental protocols. Ischemia was induced for 30 minutes, 2 hours after the *in vitro* placement of the isolated brain. NC were perfused for 1 h immediately either after the reopening of the vessel or 1 our later (protocol 2). The perfusion was followed by a wash-out period with a solution without NCs. At 5 hours *in vitro* the brains were fixed for immunohistochemistry. The bottom of the panel shows an example of simultaneous DC recordings from 4 different sites in an isolated guinea pig brain. Hypoxic depressions (HD) were recorded in the electrodes located in the regions vascularized by the occluded MCA. Potentials evoked by LOT stimulation before and during the first part of ischemia (arrowhead) disappeared when HD occurred, and recovered during MCA reperfusion. Evoked potentials in the hemisphere contralateral to MCA occlusion were not altered.

Experiments were performed on 28 in vitro isolated guinea pig brains. Transitory cerebral ischemia was induced in 25 brains by reversible, unilateral ligation of a middle cerebral artery (MCA). After MCA reopening, NCs were perfused according with the two protocols showed in the upper panel of Figure 1 (n = 19 protocol 1, n = 6 protocol 2; see Methods). In control experiments (n = 3) no occlusion of MCA was performed and NCs injection followed the time of perfusion described either in the protocol 1 (n = 2) or protocol 2 (n = 1). In all experiments, continue electrophysiological recordings were performed before and during ischemia, and during reperfusion and NCs perfusion. LOT-evoked field potentials in both PC and OT disappeared in the ischemic hemisphere 4.7±0.3 minutes (mean±SEM; n = 10) after MCA occlusion and slowly recovered at the end of the ischemia (see [14]. No recovery or partial recovery of the LOT-evoked responses was recorded in PC (n = 9), in l-OT (n = 6), in EC (n = 5), m-OT (n = 3) in 9 experiments in which post-ischemic edema developed. No changes in the morphology of the LOT-evoked field potentials was observed in the hemisphere contralateral to MCA occlusion and in control experiments (not shown). The abolition of the LOT-evoked responses was associated to large amplitude DC shifts (18.1 mV±3.6 mean±SEM; n = 10) in extracellular potentials, referred to as hypoxic depressions (HD [22]–[23]–[24]–[25]. As illustrated in Figure 1, after MCA occlusion HD developed in brain regions served by MCA, such as PC (n = 25), OT lat (n = 20) and EC (n = 9, not shown), but not in medial OT and in the PC contralateral to MCA occlusion. HDs recovered, either completely or partially, after MCA re-opening.

**Figure 2:**
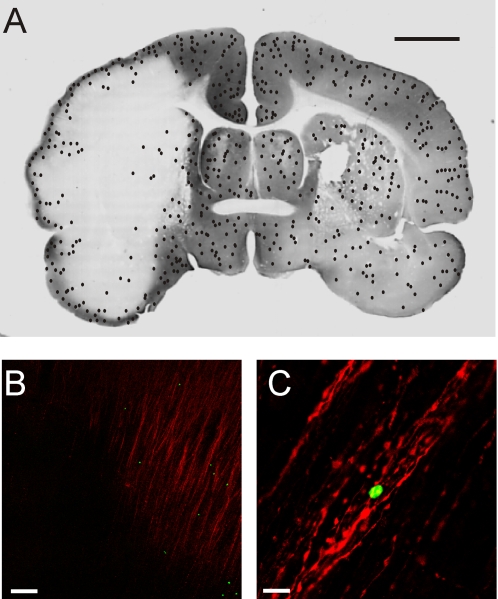
A. Recostrution of the distribution of NCs superimposed on a coronal section immunoreacted with anti-MAP-2 antibody (immunoperoxydase staining) to identify the ischemic region. Green fluorescent CFDA-stained stem cells were counted and plotted on the adjacent section stained with the MAP-2 fluorescent antibody (calibration bar = 1 cm). In B and C, microphotographs of green fluorescent CFDA-stained stem cells on sections counterstained with the fluorescent anti-MAP-2 antibody are shown at ×10 (B) and ×40 (C) magnifications. The pictures were taken in the transition region between the MAP-2^+^ and the MAP-2^−^ areas in the olfactory region. Calibration bars: 150 µm and 16 µm for B and C, respectively.

**Figure 3:**
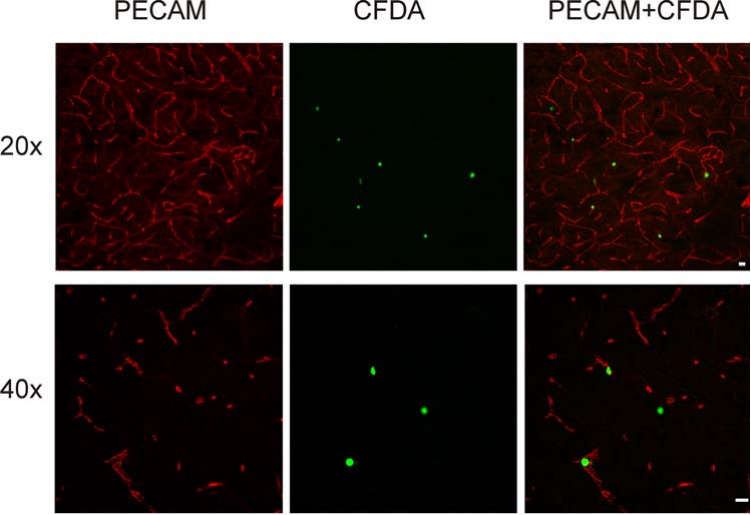
Confocal fluorescence microscope images of PECAM-1 stained sections (50 µm thick) cut from the piriform region of brains fixed at the end of the electrophysiological experiment. CFDA green fluorescent NCs and brain vessels are shown at different magnifications. Calibration bar = 100 µm. NCs were observed in close proximity to cerebral vessels.

After the electrophysiological experiment, 18 brains were processed for MAP-2 immunostaining [14], in order to identify and correlate the extension of the ischemic MAP-2 negative zone with the distribution of the fluorescent-dye-loaded NCs. The green-fluorescent NCs were counted in the MAP-2 positive and MAP-2 negative zones of the control and ischemic hemispheres (n = 6). In 6 experiments cell counts were not performed because NCs clustered in long chains into vessel bifurcations, suggesting the formation of clogs during arterial perfusion. Experiments in which edema developed and the tissue was consequently poorly fixed, were also excluded by the NCs counts (n = 6). A representative pattern of distribution of NCs is shown in the experiment illustrated in Figure 2. The distribution and numbers of NCs were similar in protocol 1 and 2. Table 1 shows the mean NCs densities (cells/cm^2^) in different experiments (n = 6) in three regions of interest defined below. Even though the absolute number of the fluorescent NCs observed in the brain was variable among the experiments, their relative distribution in the MAP-2-positive and MAP-2-negative areas was reproducible across experiments. Statistical significance was observed between cell densities in the control hemisphere MAP-2**^+^** area and the ischemic MAP-2**^−^** region (p<0.05), and between the MAP-2**^+^** and MAP-2**^−^** areas ipsilateral to the MCA occlusion (p<0.03). No significant differences were observed between NCs counts in MAP2 positive areas of control hemispheres (Wilcoxon test; n = 6). In sections immunoreacted with an antibody against the endothelial adhesion molecule, PECAM-1, merged confocal images of high magnification (20×, 40×) show cells in close proximity to brain vessels (Figure 3).

**Table 1:** Mean NCs density (cells/cm^2^) in the MAP-2 positive zone contralateral (left column) and ipsilateral (middle column) to the ischemic side and in the MAP-2 negative zone of the ischemic hemisphere (right column) in 6 different experiments.

	Control hemisphere	Ischemic hemisphere	
	MAP+	MAP+	MAP−
**Exp. 1** (n = 5)	74.25±4.9/cm2	60.25±4.2/cm2	2.25±0.8/cm2
**Exp. 2** (n = 4)	565.6±121/cm2	440±44.1/cm2	119.4±27.4/cm2
**Exp. 3** (n = 3)	93.3±8.7/cm2	57±6.3/cm2	60±17.5/cm2
**Exp. 4** (n = 3)	963±201/cm2	845±118/cm2	294±11/cm2
**Exp. 5** (n = 3)	878±68/cm2	480±121/cm2	53±5/cm2
**Exp. 6** (n = 5)	617±92/cm2	777±98/cm2	244±37/cm2

On the left, the number of slices on which counts were made for each experiments is indicated.

**Figure 4:**
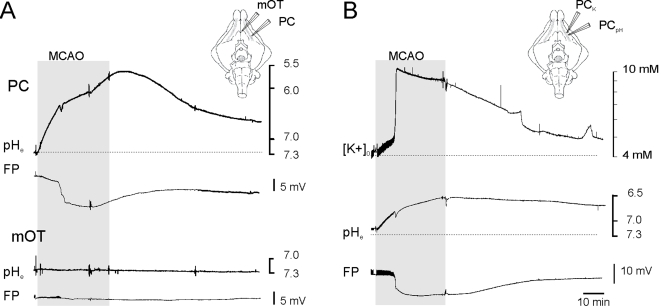
(A) Changes in extracellular pH (pH_e_) in the PC and in mOT induced by ipsilateral MCA occlusion and reperfusion. Occlusion induced a rapid metabolic acidification of the extracellular microenviroment in PC, interrupted by a transient and mild basification (arrow) associated to the hypoxic spreading depression (HD, asterisk). No changes in extracellular [H^+^] were recorded in the mOT, that is not served by the MCA. (B) Simultaneous changes in extracellular potassium concentration ([K^+^]_o_)and extracellular pH in the PC after MCA occlusion and reperfusion. An initial enhancement in [K^+^] was followed by a fast and large increase in [K^+^]_o_. associated to the HD. The schematic drawing on the right illustrates the position of the two-barrel recording electrodes. The field responses (FP) recorded with the conventional extracellular barrel are also shown. The period of MCA occlusion is marked by the shaded area.

We conclude that NCs less likely stop into the core of the ischemic MCA region. Unfavorable local tissue changes could be responsible for this phenomenon. In order to study extracellular changes correlated with brain ischemia, pH measurements were carried out with ion-sensitive electrodes in PC and m-OT ipsilateral to the MCA occlusion and, as control, in the contralateral PC. As shown in Figure 4A, MCA occlusion was associated with a rapid metabolic acidification of the extracellular microenvironment (1.44±0.29 pH units measured at peak values; n = 8) briefly interrupted by a transient and mild basification (arrow) that coincided with a sharp negative shift associated to the hypoxic depression (HD; see also [14]. The recovery of HD was paralleled by a slow increase of extracellular pH that only in few experiments reverted to pre-ischemic values. No significant changes in extracellular pH were recorded in those areas not involved in cerebral ischemia (0.1±0.04; mean±SE; n = 5).

To better characterize the extracellular microenvironment changes associated with the ischemic process, simultaneous pH and potassium measurements ([K^+^]_o_) were performed in the PC of the ischemic hemisphere (n = 2). When MCA occlusion was performed, a gradual increase in [K^+^]_o_ was observed (Figure 4B). The ischemic depressions observed with the conventional electrode invariably determined a fast and large increase in [K^+^]_o_, that returned toward pre-ischemic values upon MCA territory reperfusion.

## Discussion

The present report demonstrates that NCs injected through the vascular system of the *in vitro* isolated guinea pig brain after transitory occlusion of the MCA mainly distribute in areas spared by the ischemic injury. The ischemic area in the MCA territory was defined by using MAP-2 staining, a reliable marker of dendro-somatic anoxic damage that rapidly decreases in response to acute tissue injury because of microtubules proteolysis [26]–[27]–[28]. In our experiments, the MAP-2 immuno-negative region was identified as the core of the ischemic injury, while the surrounding areas characterized by clustered chains of immunoreactive product were identified as potential areas of ischemic penumbra in watershed regions between the MCA and anterior cerebral and limbic arteries [14]–[29]. In addition to MAP-2 immunohstochemical staining, complementary electrophysiological parameters, such as the disappearance of evoked potentials and the occurrence of slow extracellular DC shifts were utilized to identify the ischemic region the *in vitro* isolated guinea pig brain.

Our findings suggest that NCs homing into the ischemic tissue is impaired during the very initial phase of the ischemic process, in spite of the presumed induction of chemotactic signals associated to the acute tissue damage [30]–[31]–[32].This finding is corroborated by in vivo studies that showed low numbers of NCs in the ischemic core 3–6 hours after intravenous injection [11]. Unlike white blood cells [33]–[34], which require an appropriate level of endothelial activation/inflammation to adhere to cerebral vessels in the isolated guinea pig brain [20], NCs showed the ability to stop into the vessels of control brains and into non ischemic areas of brains in which MCA occlusion was performed. The arrest of NCs in brain tissue is possibly due to membrane expression of various surface molecules that promote brain vessel adhesion [11]–[35]. The low number of NCs observed in the ischemic core is probably not due to the loss of their ability to adhere to vessels and transmigrate, but rather to their damage consequent to the unfavorable local micro-environmental changes that occur into the cerebral parenchyma during the acute phase of ischemic injury. In the acute phase of ischemia, indeed, release of excitotoxic neurotransmitters, free radicals, and pro-inflammatory mediators and changes in tissue pH might threaten blood-borne cells introduced into the peri-infarct region [36]. Moreover, post-ischemia reperfusion triggers barrier permeability changes and alterations of endothelial compartment that affect microvascular integrity [37]. In such a compromised microenvironment, the relatively low probability of arrest of NCs into brain vessels is not so surprising, at least in the early minutes/hours that follow the ischemic event. Interestingly, preliminary observations demonstrate that other cell types, such as activated lymphocytes and polymorphonucleates that adhere to inflamed endothelium in the isolated brain preparation, tend to avoid the ischemic core in our experimental ischemic conditions.

A prominent and consistent change observed in our experiments during ischemic brain tissue was a gradual extracellular acidification that reflects acid production and accumulation of CO_2_ [38], The pronounced, progressive and large extracellular acidification induced by MCA occlusion correlated to HD events characterized by large amplitude extracellular DC shifts coupled with synaptic activity depression and large [K^+^]_o_ increases [14]–[39]–[40]–[41]. Tissue acidosis has been suggested to be a pathogenic factor for the development of cytotoxic brain edema and cell death. When protracted, brain tissue acidosis accelerates catalytic processes that lead to neuronal, glial and microvessels destruction [37]–[42]–[43]–[44]. Acidosis leads to functional changes of endothelial cells [45] that, in principle, could either hinder NCs homing or damage NCs migrated into the ischemic core region.

According with previous data recently published by our group [14] , we can reasonably exclude that an impaired post ischemic arterial perfusion could account for the accumulation of NCs in non-ischemic areas. As discussed in the mentioned study, the effect of transient MCA occlusion on the evoked response reversed 15 minutes after re-perfusion, confirming a restored oxygenation of all territories supplied by the MCA, including the area identified as ischemic “core”. Moreover, NCs were able to stop into the ischemic core when no cell wash-out was performed (Librizzi, Pastori and de Curtis, unpublished observations). This observation further strengthen the idea that local micro-environmental changes occurring into the ischemic core are responsible for NCs homing into the penumbra area.

The issues of the eventual fate of the injected NCs and their effects on the outcome of cerebral ischemia were not addressed in this study. No differences in the extension of the ischemic region measured with both electrophysiological and immunohistochemical means was observed when NCs were applied, in comparison to experimental conditions in which MCA occlusion was not followed by NCs injection. Considering the short time window of our observation (max 3 hours after the induction of ischemia), we cannot exclude that NCs could migrate in a later phase from the MAP-2 positive penumbra regions towards the MAP-2 negative ischemic zone. In experimental models of CNS inflammation, there is solid evidence that precursor neural cells survive local intracerebral transplantation and migrate specifically into the damaged tissue after MCA occlusion [46]. In these experiments, the functional recovery of the ischemic region determined by NCs treatment scarcely correlated with absolute numbers of transplant-derived cells observed in the brain, that greatly decreased within few days after transplantation in spite of the positive functional outcome. Therefore, it cannot be excluded that NCs could be therapeutically efficacious through a bystander mechanism possibly mediated by the release of neuroprotective factors in the peri-ischemic tissue [13]–[47]. This recently described hypothetical mechanism does not require that stem cells replace damaged neurons by assuming neuronal or glial phenotype in the peri-ischemic brain area, as previously suggested by experiments of intracerebral transplantation of human fetal neural stem cells in murine models of MCA occlusion [8]–[11]. Capone and colleagues observed, indeed, the gradual disappearance of NCs within the first week after localized intracerebroventricular stem cell injection, in spite of the neuroprotective effect evaluated by quantifying functional impairment and neuronal damage 1 week and 4 weeks after the ischemia in comparison with sham control animals.

### Conclusions

Our data show that NCs reach the ischemic-injured cerebral area but preferentially distribute outside the ischemic core, in non-ischemic regions. We propose that early microenvironment changes that follow the occlusion of the MCA could explain this phenomenon, and speculate that the beneficial effect of NCs in focal brain ischemia is not strictly dependent on their homing into the ischemic region.

## Materials and Methods

### Brain isolation and transient focal ischemia protocol

Brains of young adult Hartley guinea-pigs (150–250 g weight, Charles River Laboratories, Comerio, Italy) were isolated and maintained *in vitro* according to a previously described technique [16]–[17]–[48]–[49]. Brain isolation was performed at low temperature (15°C) and brains were perfused *in vitro* at 7 ml/min via the resident arterial system with a complex saline solution composed of 126 mM NaCl, 3 mM KCl, 1.2 mM KH_2_PO_4_, 1.3 mM MgSO_4_, 2.4 mM CaCl_2_, 26 mM NaHCO_3_, 15 mM glucose and 3% dextran M.W. 70.000, oxygenated with a 95% O_2_-5% CO_2_ gas mixture (pH 7.3). Experiments were performed at 32°C. Transient focal ischemia was induced by unilateral ligation of the middle cerebral artery (MCA) for 30 minutes following the protocol previously described [14]. Briefly, a silk thread node was prepared around the proximal portion of the MCA. Two hours after *in vitro* placement, the MCA was closed by pulling both ends of the thread; the node was re-opened after 30 minutes by releasing the thread. The experimental protocol was reviewed and approved by the Ethics Committee of the Istituto Neurologico, according to National and International guidelines on care and use of laboratory animals (protocol number 45–46; 07/11/2003).

### Neural Stem Cell preparation and perfusion in the isolated brain

Neurosphere-derived cells (NC) were isolated from newborn mice C57BL/6 (Charles River, Calco, Italy). The brain of each mouse was removed and mechanically triturated with a fire-polished Pasteur pipette. The tissue suspension was collected and treated with collagenase Type I (Gibco, Paisley Scotland, U.K.) and DNAase I (USBiological DBA Italia, Italy) for 30–45 minutes at 37°C in a humidified atmosphere containing 5% CO_2_. Pellets were dissociated in a 200 µl pipette tip, plated in an untreated 25 cm^2^ tissue culture flasks and cultured in medium optimized for NCs growth in neurospheres [50] Neurospheres were harvested and dissociated at single cells with collagenase I and re-plated under the conditions described above. After performing this procedure twice in order to eliminate short-term dividing precursors, bulk cultures were generated by passing the cells at higher density in the same growth medium every 10 days. Neurospheres cultures can be routinely propagated up to 20 passages. They can be induced to differentiate into astrocytes, neurons and oligodendrocytes by culturing in serum and growth factors-free medium plus 2% fetal bovine serum.

Cells for transplantations were used starting from the 7^th^ passage. After dissociation, cell count and viability test were assessed by Trypan blue exclusion. Single suspension of NCs were stained with warm 7.5 µM green fluorescent dye Vybrant CFDA (S.E. Cell Tracer kit V-12883; Molecular Probes, Eugene, OR, USA) for 15 minutes at 37°C in a humidified atmosphere containing 5% CO_2_ and then re-suspended in phosphate buffer saline (PBS) without calcium and magnesium. The absolute number of cells was evaluated by light microscopy. Vitality of NSCs before and after fluorescent staining was consistently evaluated in each experiment by Trypan Blue staining in Fuchs-Rosenthal chamber. NSCs with vitality ≥85% were used in each experiment. NCs (1×10^6^ in 5 ml of PBS) were infused into the perfusion line at a 0.08 ml/min rate with an injection pump (KD Scientific type 250, USA) either just after (protocol 1 in Figure 1) or 1 hour after MCA re-opening (protocol 2 in Figure 1). NC perfusion was followed by wash-out with a cell-free solution.

### Electrophysiological recordings

Extracellular direct-current (DC) recordings were continuously performed before, during and after MCA transitory occlusion and during the NCs perfusion and washout. Single glass pipettes filled with 0.9% NaCl were positioned in the anterior piriform cortex (PC; n = 25) of both hemispheres, in the medial (n = 23) and lateral (n = 22) olfactory tubercle (OT) and in lateral entorhinal cortex (EC; n = 10) on the same side of MCA occlusion (see schematic drawing in Figure 1). The position of the recording electrodes was visually controlled with a stereoscopic microscope. Simultaneous extracellular recordings were performed in different combinations with a maximum of 5 recording sites. The lateral olfactory tract (LOT) was stimulated with a twisted silver wire to evaluate evoked responses in the olfactory-limbic cortices (see Biella et al., 2000). Electrophysiological signals were amplified via a multichannel differential amplifier (Biomedical Engineering, Thornwood, NY) and were acquired utilizing the software developed by Dr. Vadym Gnatkovsky in our laboratory (ELPHO™).

### Ion-selective electrodes and recordings

Two-barrels, ion-selective electrodes (tip diameter 3–5 µm) were utilized to record field responses simultaneously with either extracellular potassium ([K^+^]_o_) or extracellular pH in PC and in medial OT [19]. For [K^+^]_o_ recordings, the conventional electrode was filled with KCl 10 mM. The barrel utilized for [K^+^]_o_ measurements was filled at the tip with the potassium ionophore I cocktail A (Fluka 60031, Germany) after 1 minutes exposure to dimethyldichlorosylane vapors (Fluka, Germany) and was backfilled with 10 mM KCl following a 2 h incubation at 120°C. Potassium calibration solutions had the same composition of the solution used for arterial perfusion, except for KCl concentration, which was modified to obtain final K^+^ concentration of 1, 2.5, 6, 12.5 and 48 mM. Only microelectrodes with a response of 30–40 mV for 10 mM of K^+^ were utilized. Proton-sensitive double barreled electrodes were also utilized to measure extracellular pH. The procedure to prepare pH-sensitive electrodes was similar to the protocol for K^+^-sensitive electrodes. The tips of the ion-sensitive barrel were filled with hydrogen ionophore II-cocktail A (Fluka 95297) and back-filled with a buffer solution (in mM: NaCl 100, HEPES, and NaOH 10, pH 7.5). For the pH-electrode calibration, the pH of the perfusate was set between 5.5 and 7.5. The pH-ion sensitive electrodes had a response of 50–55 mV for unit change in pH. Ion-selective and field DC signals were amplified with a high-input impedance head-stage amplifier (Biomedical Engineering, Thornwood, NY). Subtraction of the field potential from the ion-sensitive electrode voltage reading was performed. Data were acquired by the same software utilized for single glass pipettes recordings.

### Immunocytochemistry

Five hours after the *in vitro* placement, brains were fixed by immersion in a 4% paraformaldehyde solution in phosphate buffer (PB; 0.1 M, pH 7.4) for one day. Brains were then cut in serial coronal slices (50 µm thick) with a vibratome (VT 1000S; Leica Heidelberg, Germany) throughout their rostro-caudal extension. Slices were immunoreacted for microtubule associated protein, MAP-2 [28]–[51]–[52], utilized as a marker of the ischemic brain area [14]–[26]–[27]–[28] and for anti-platelet endothelial cell adhesion molecule-1, PECAM-1 [41]; 1∶100) to outline cerebral vessels [53]. Selected free-floating sections at 3 different levels rostral to the optic chiasm were collected in pairs and were alternately processed for either immunoperoxidase or immunofluorescent labeling. For immunoperoxidase staining, sections were pre-incubated for 10 minutes in 3% H_2_O_2_ in phosphate-bufffered saline (PBS) pH 7.4, to inactivate endogenous peroxidase and were then rinsed in PBS. Nonspecific sites were blocked in PBS with 10% normal horse serum (NHS) and 0.2% Triton X-100. Sections were incubated overnight with anti-MAP-2 antibody at 4°C (1∶1000 MAP-2 clone AP 20, Bio-Optica, Fremont, CA, USA) diluted in 1% NHS in PBS. After several rinse in PBS, sections were then incubated for 75 minutes in PBS with biotinylated horse anti-mouse IgG (1∶200; Vector Laboratories Inc., Burlingame, CA, USA). The avidin-biotin peroxydase protocol (ABC kit, Vector Labs) was applied, using 3,3′-diaminobenzidine tetrahydrochloride (DAB; Sigma, St. Louis, MO, USA) as chromogen. Slices were mounted, dehydrated, cleared with xylene and coverslipped with DPX.

For immunoflorescence-staining, alternate sections at each selected rostro-caudal level were incubated first for 1 h in PBS containing 10% NHS and 0.2% Triton X-100 and then overnight in the primary antibody MAP-2 diluted 1∶1000 in PBS with 1% NHS at 4°C. Slices were incubated for 2 h at room temperature in indocarbocyanine (CY3)- conjugated horse-anti-mouse IgG (1∶600, Jackson Immunoresearch Labs, West Grove, PA, USA). After further rinsing in PBS, the sections were mounted in Fluorosave (Calbiochem, San Diego, CA, USA) and were examined under a confocal laser scanning microscope (Bio-Rad, Hemel Hemstead, UK) equipped with an argon/krypton gas laser mounted on a light microscope (Eclipse E 600; Nikon, Tokio, Japan). CY3 was excited at 550 nm lines. NCs, stained with green fluorescent dye Vybrant CFDA, were exicited at 490 nm lines. Confocal image series micrographs were collected and digitized as TIFF files with a Radiance 2000 confocal scanning microscope and software package.

### Quantitation of green-fluorescent NCs and statistical analysis

The green-fluorescent NCs were counted in each hemisphere and were quantified in the MAP-2 positive and MAP-2 negative zones of the ischemic hemisphere and in the MAP-2+ control hemisphere contralateral to the ischemic site. Three to seven sections at different rostro-caudal levels were immunoreacted after the electrophysiological experiment (n = 6). In at least 3 representative slices per experiment, the fluorescent SCs were counted on confocal microscope in both hemispheres. In 6 experiments, using Imaging pro Plus 5.1 software (Media Cybernetics, MD, U.S.A) MAP-2 positive and of MAP-2 negative areas were calculated on DAB sections just adjacent to those utilized for fluorescent counts. Wilcoxon test was performed to analyze data significance.
